# Supplementary material for: Charge transfer as a ubiquitous mechanism in determining the negative charge at hydrophobic interfaces
Source: Nat Commun. 2020 Feb 14;11:901. doi: 10.1038/s41467-020-14659-5 (PMC7021814; doi:10.1038/s41467-020-14659-5)
Supplement: Supplementary file 1 — Supplementary Information [file 41467_2020_14659_MOESM1_ESM.pdf]

Charge Transfer as a Ubiquitous Mechanism in Determining the Negative Charge at Hydrophobic Interfaces

E. Poli et al.*

E-mail: epoli@ictp.it,ahassanali@ictp.it

Supplementary Methods

The results we reported in the main text were all performed with the BLYP+D2 functional and the DDEC charge partition scheme. We have performed a series of validation and benchmark tests in order to examine the sensitivity of our results with respect to the choice of DFT functional, as well as looking at different charge partitioning schemes (in the main text) and sampling protocols. Specifically, we wanted to assess

Air-Water Interface

For the water/air interface, 20 frames were picked randomly out of the 250 considered in the main text. The charge density, as shown in the left panel of Figure 2 in the main text, was recomputed using a different protocols: rVV10[2] non-local, van-der-Waals functional with DDEC charges, BLYP+D2 with Iterative Hirshfield charges, BLYP+D2 with Iterative Stockholder Analysis charges and BLYP+D2 with NBO charges. We also performed some more expensive B3LYP hybrid functional calculations with DDEC charges using only 4 frames. The B3LYP calculations were performed with the CP2K code using Goedecker-Teter-Hutter (GTH)[3] norm-conserving pseudopotentials to model the electron-ion interactions. We adopted the molecularly optimized (MOLOPT) TZVP-SR basis set[4] for all atoms and the auxiliary density matrix method (ADMM)[5] to compute the exchange integrals, using the cpFIT3 basis set for all the elements present in our systems. All these various combinations of density functional and charge schemes are shown in Figure 8 in the main text and Supplementary Figure 1 here. We see that the charge layering at the air-water interface is consistently reproduced regardless of the functional used. Supplementary Figure 2 shows the scatter plots comparing the DDEC charges obtained from BLYP-D2 with respect to those obtained with NBO and IH charge schemes (from the main text), the rVV10 functional and finally the B3LYP hybrid functional. Given the delicate role that the classical potential of choice plays in determining the structure of the interfacial water at the water-air boundary we also tested the validity our results against structures obtained using the mb-POL potential. MB-pol is a "first principles" water potential with flexible

monomers for molecular simulations of water systems from gas to condensed phases. MB-pol explicitly treats the one-body term and the short-ranged two- and three-body terms. MB-pol can thus be viewed as a classical polarizable potential supplemented by short-range two- and three-body terms that effectively represent quantum-mechanical interactions arising from the overlap of the monomer electron densities. MB-pol does not use any empirical parameter and nonetheless accurately describes the properties of gas-phase clusters, such as the dimer vibration-rotation tunneling spectrum, second and third virial coefficients, cluster structures and energies. In addition path-integral molecular dynamics (PIMD) and centroid molecular dynamics (CMD) have shown that MB-pol gives a highly accurate description of the liquid phase of water at ambient conditions in comparison with experiment for several structural, thermodynamic, and dynamical properties. 10 frames were extracted randomly by a 10 ns NVT molecular dynamics from a box measuring 26.0 x 26.0 x 100 Åcontaining 512 H_2O molecules. LS-DFT calculations with the same parameters indicated in the main text were run for these systems and the DDEC charges extracted.

Oil-Water Interface

One of the important findings of our contribution, is the charge transfer between water and oil leaving the latter negatively charged. Similar to the water/air case 10 frames were picked randomly out of the 200 considered and were recomputed using the VV10 functional and the different charge schemes listed for the air/water case. In addition, in order to assess the sensitivity of our results to the choice of a hybrid functional (B3LYP) and wave function (MP2) based approaches, we performed some benchmarks on smaller clusters carved out from the thermal simulations described in the main text. The clusters typically consist of about 12 H_2O molecules and one decane molecule and are the same used for the EDA analysis reported in the main text. The calculations on the clusters were run with CP2K using the same parameters described above for the B3LYP case. For the MP2 calculations, the cc-TZV2P basis-set[6] together with the corresponding Goedecker-Teter-Hutter-type pseudo-potentials[3]

have been adopted for all the calculations. In addition the RI-JK TZ-fiodena-opt auxiliary basis set was used for the RI-MP2 part of the calculations[6]. HF exchange calculations have been performed using the Γ-point implementation, the Schwarz screening threshold for was set to 10^{-10} for all the clusters.The truncated Coulomb operator (for Hartree-Fock exchange) was applied using one-half the length of the smallest edge of the simulation cell as truncation radius. The threshold for the SCF convergence was 10^{-7} or tighter. The PW cutoff for the HF part of the calculations was 500 Ry. The DDEC charges were calculated on the basis of the MP2 electronic density.

Supplementary Figures

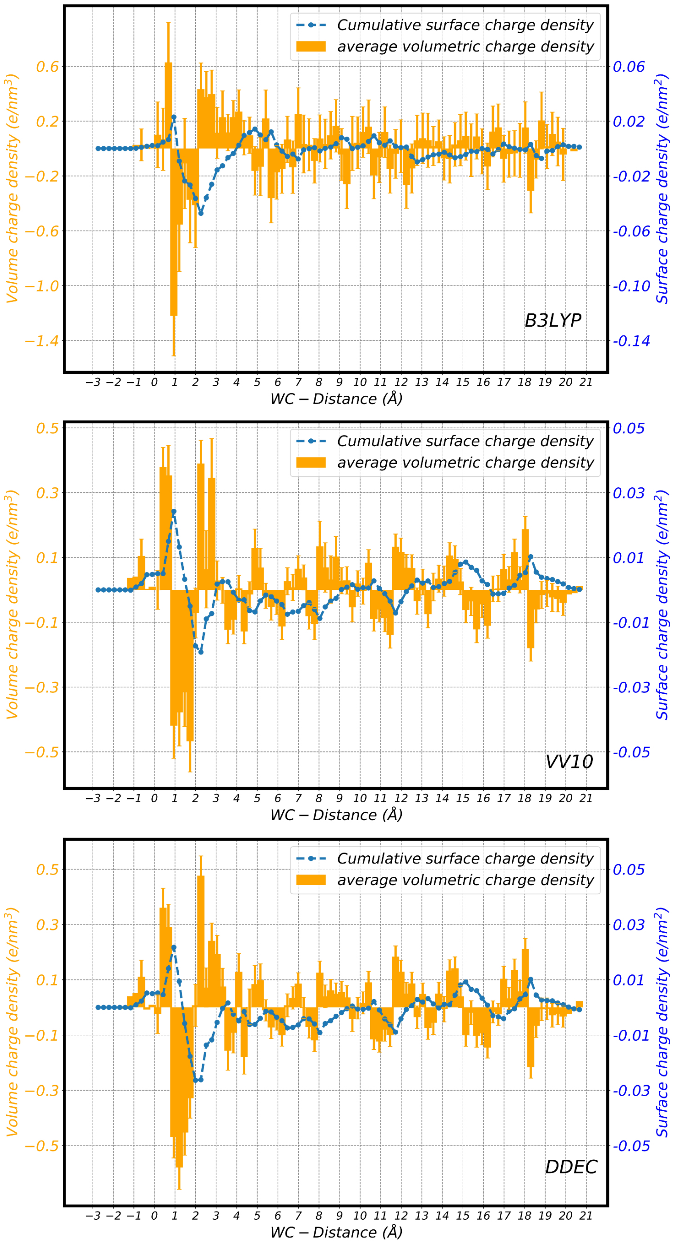

Supplementary Figure 1: Comparison of the charge profiles obtained for 20 test water/air frames (4 frames for the B3LYP case, see previous text) using DDEC charges with B3LYP, VV10 and BLYP+D2 functional (respectively upper panel, second panel and lower panel). The same labels and axis references as Figure 2 in the main text were used.

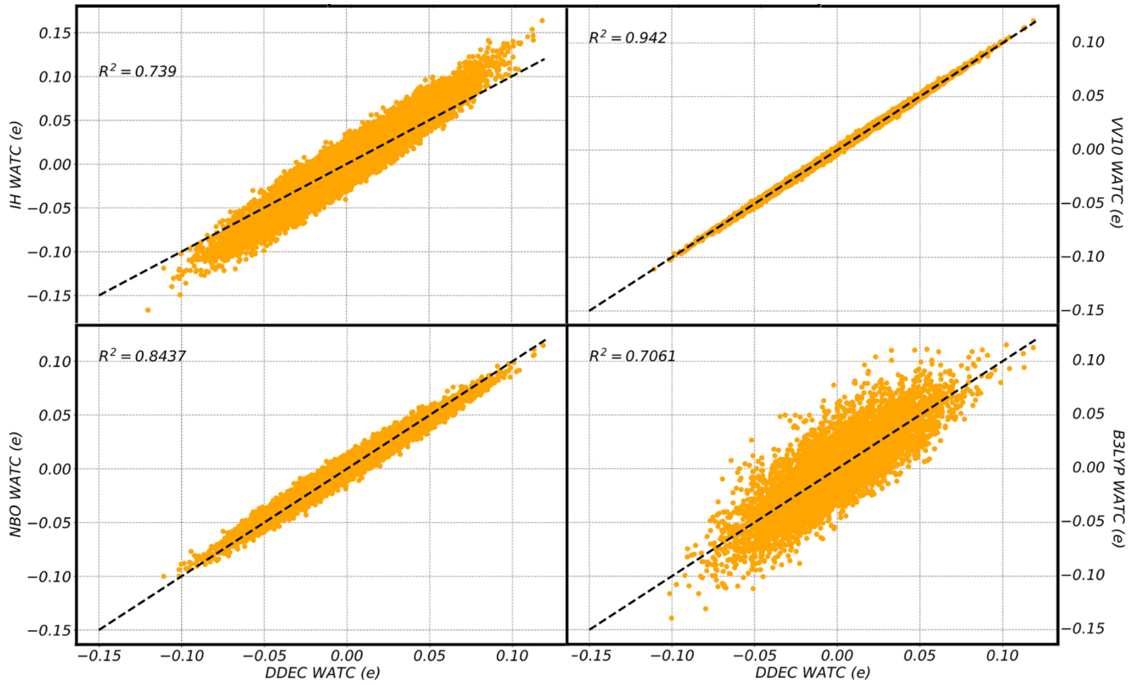

Supplementary Figure 2: One to one comparison between the water net charges calculated using different schemes IH .vs. DDEC (upper left panel), NBO .vs. DDEC (lower left panel) and different functionals VV10 .vs. BLYP+D2 (upper right panel) and B3LYP .vs. BLYP+D2, (lower right panel) for each water molecule in 20 test air/water frames randomly selected from the ones used in the main study The black diagonal represent the perfect agreement between data. The R^2 regarding the data points is also reported.

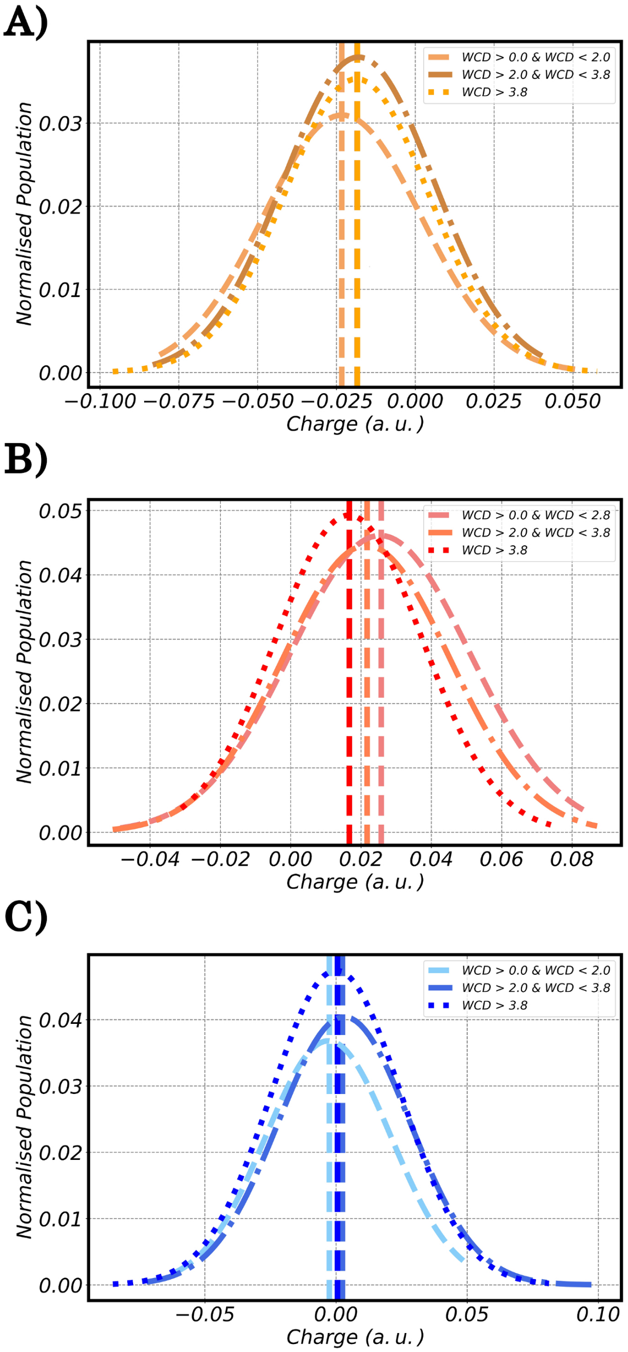

Supplementary Figure 3: Layer-by-layer charge distributions of the, 1in-2out (panel A), 2in-1out (B) and 2in-2out (C) water molecules for the MB-POL water-air interface. The first moment is highlighted by a vertical line for each distribution

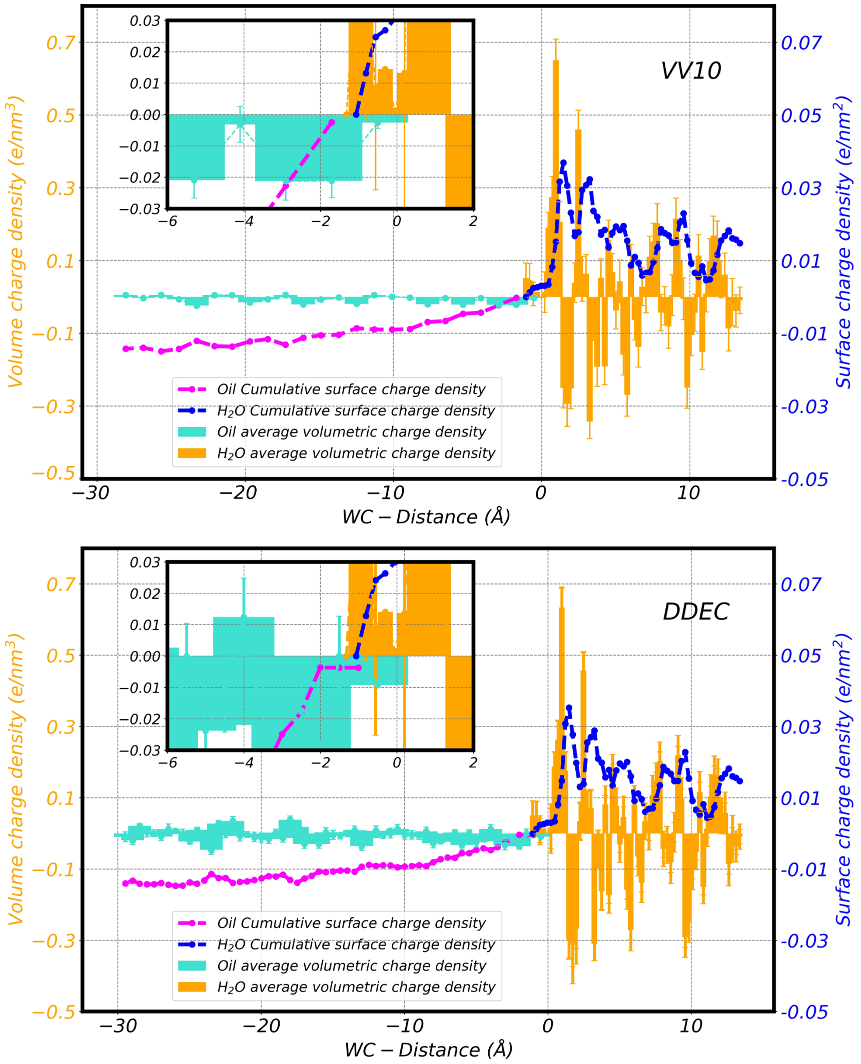

Supplementary Figure 4: Comparison of the charge profiles obtained for 10 test water/oil frames using DDEC charges (with VV10 functional, upper panel and with BLYP+D2 functional, lower panel). The same labels and axis references as Figure 2 in the main text were used.

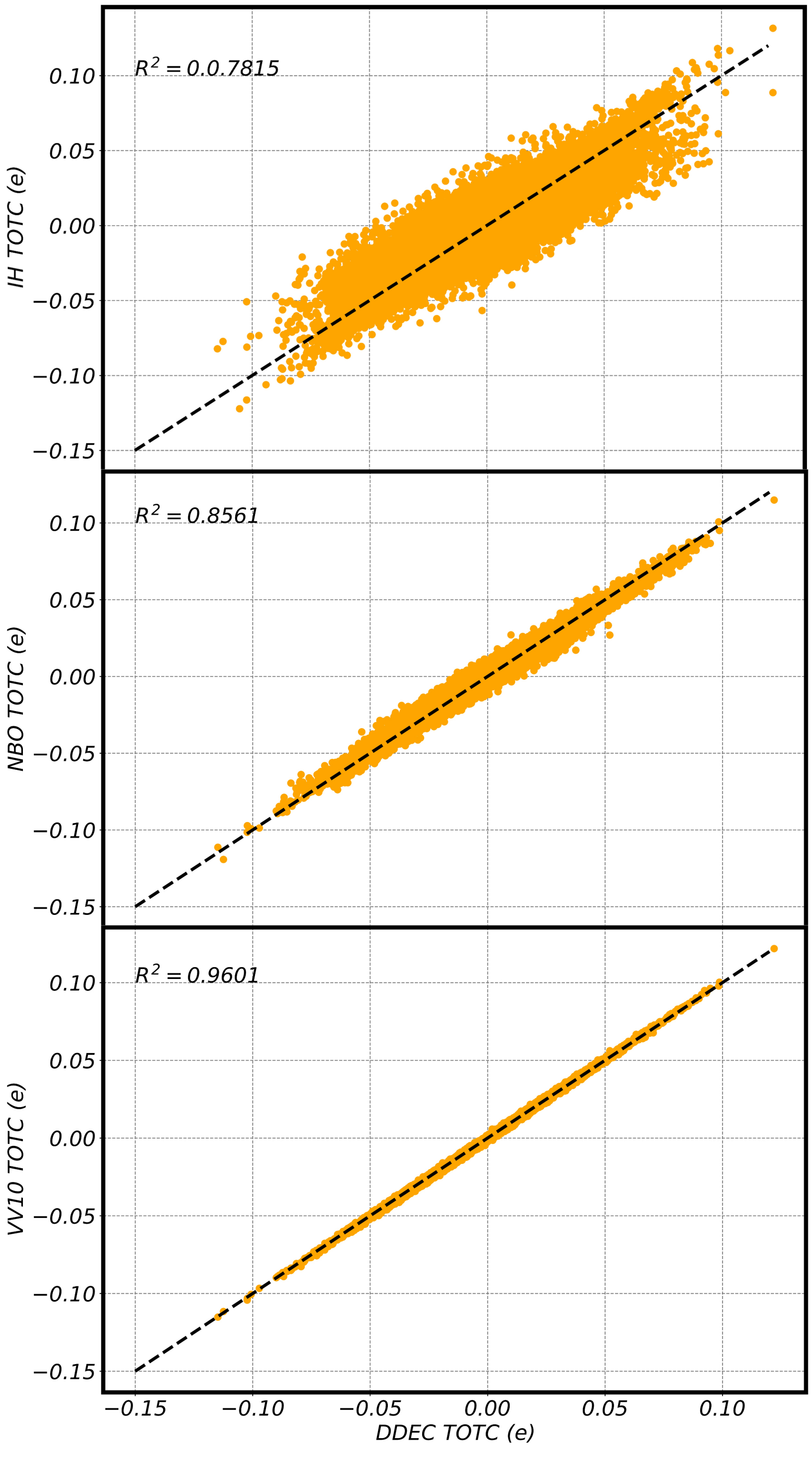

Supplementary Figure 5: One to one comparison between the water net charges calculated using different scheme IH .vs. DDEC (upper panel), NBO .vs. DDEC (second panel) and different functionals VV10 .vs. BLYP+D2 (lower panel) for each water molecule in 10 test oil/water frames randomly selected from the ones used in the main study. The black diagonal represent the perfect agreement between data. The R^2 regarding the data points is also reported.

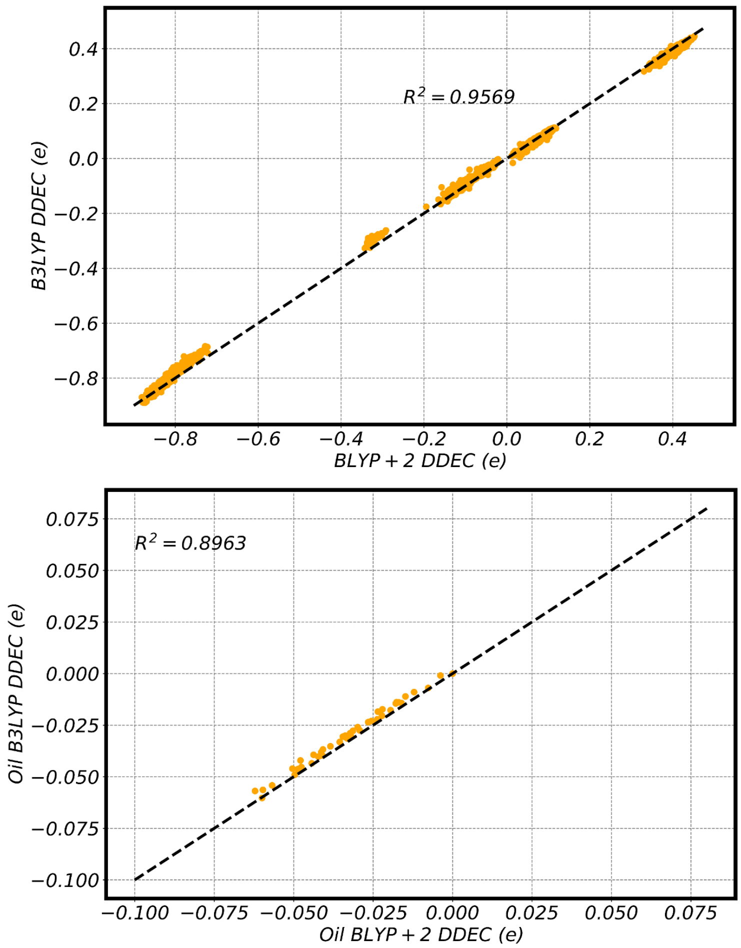

Supplementary Figure 6: One to one comparison between the DDEC charges calculated using different functionals B3LYP .vs. BLYP+D2 (upper panel), for each water/oil cluster considered in the Energy Decomposition Analysis reported in the main text. The comparison of between the net negative charges on the oil molecules is also reported (lower panel). The black diagonals represent the perfect agreement between data. The R^2 regarding the data points are also reported.

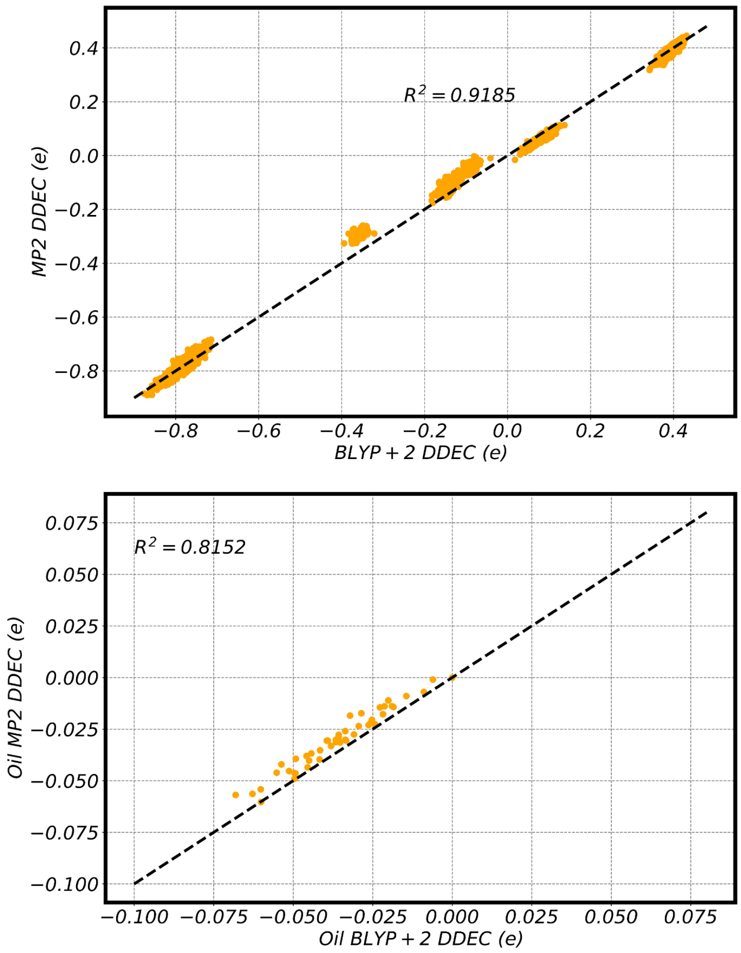

Supplementary Figure 7: One to one comparison between the DDEC charges calculated using different methods HF-MP2 .vs. BLYP+D2 (upper panel), for each water/oil cluster considered in the Energy Decomposition Analysis reported in the main text. The comparison of between the net negative charges on the oil molecules is also reported (lower panel). The black diagonals represent the perfect agreement between data. The R^2 regarding the data points are also reported.

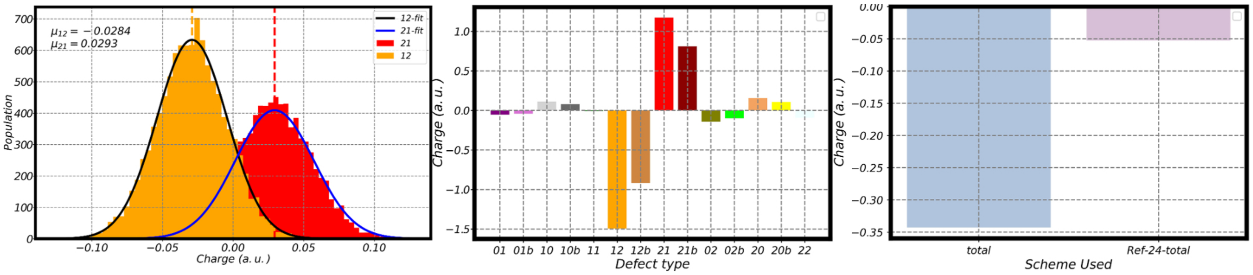

Supplementary Figure 8: Gaussian fitting of the 1in-2out (orange), 2in-1out (red) populations in the second layer (left panel). The mean is reported in the upper left part of the panel. Comparison between total charges obtained by our calculations and charges obtained applying the method in Supplementary Ref. 7 (central panel) The charges obtained using the method in Supplementary Ref. 7 are labelled with a **b** next to the species name. Net charges obtained for the second layer using ab-initio calculation and the method reported in Supplementary Ref. 7 (Right panel).

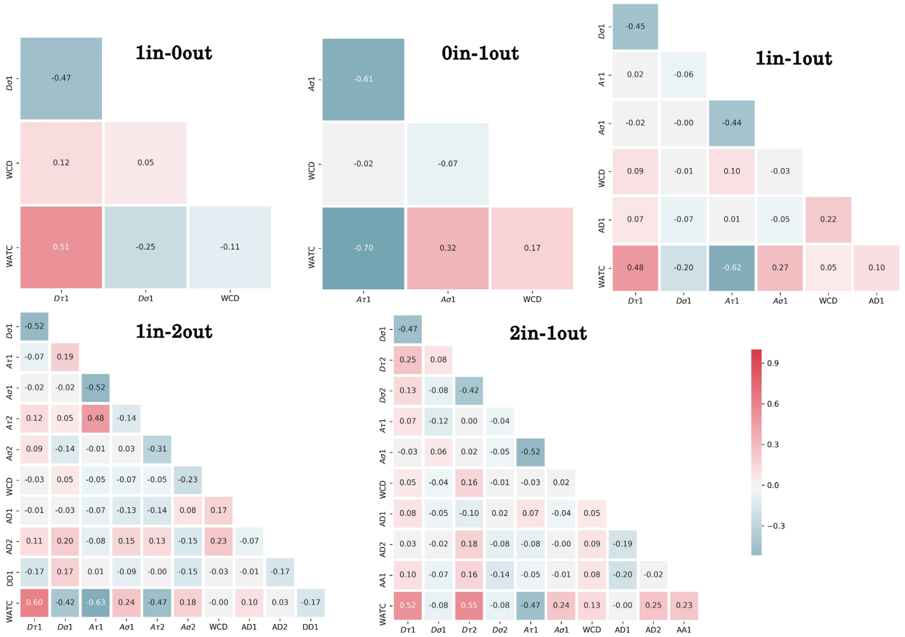

Supplementary Figure 9: Correlation matrix (using Pearson coefficients) for the first layer between the different geometrical features considered and reported in Figure 9 of the main text (Panel A and B), the distance from the Willard Chandler interface (WCD) and the water molecules total charge (WATC).

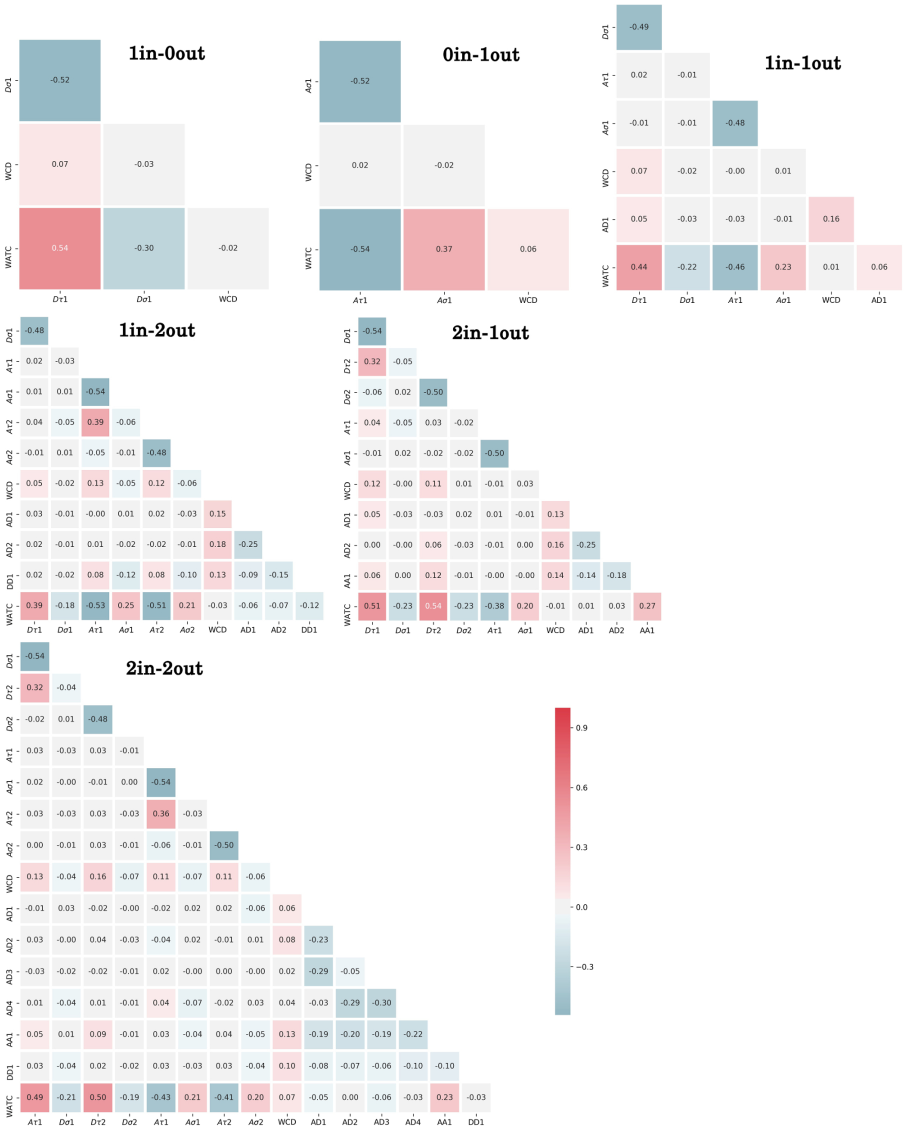

Supplementary Figure 10: Correlation matrix (using Pearson coefficients) for the second layer between the different geometrical features considered and reported in Figure 9 of the main text (Panel A and B), the distance from the Willard Chandler interface (WCD) and the water molecules total charge (WATC).

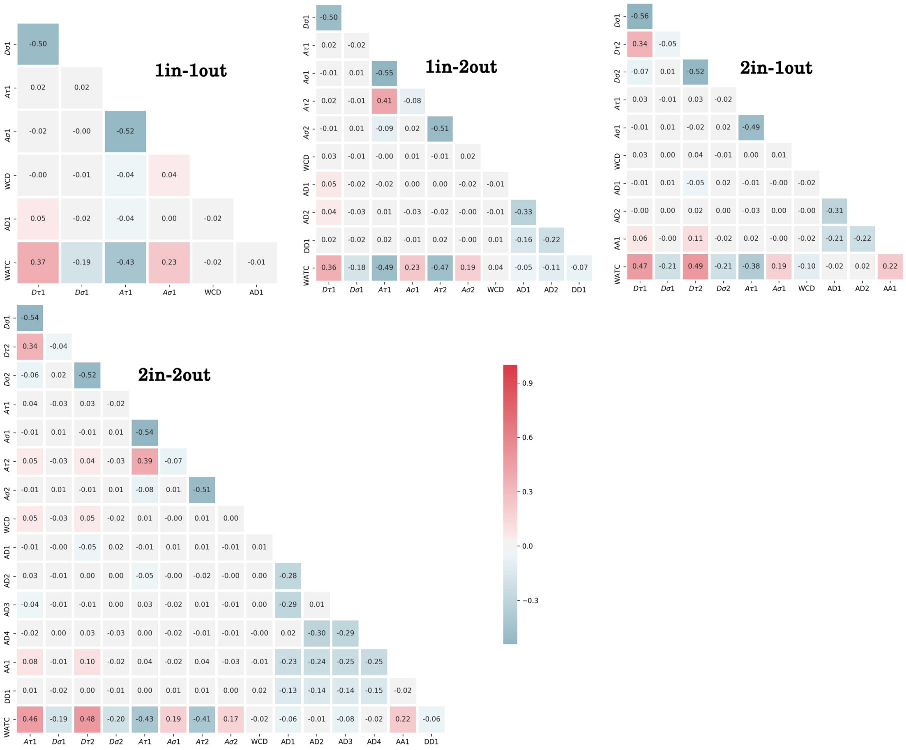

Supplementary Figure 11: Correlation matrix (using Pearson coefficients) for the third layer between the different geometrical features considered and reported in Figure 9 of the main text (Panel A and B), the distance from the Willard Chandler interface (WCD) and the water molecules total charge (WATC).

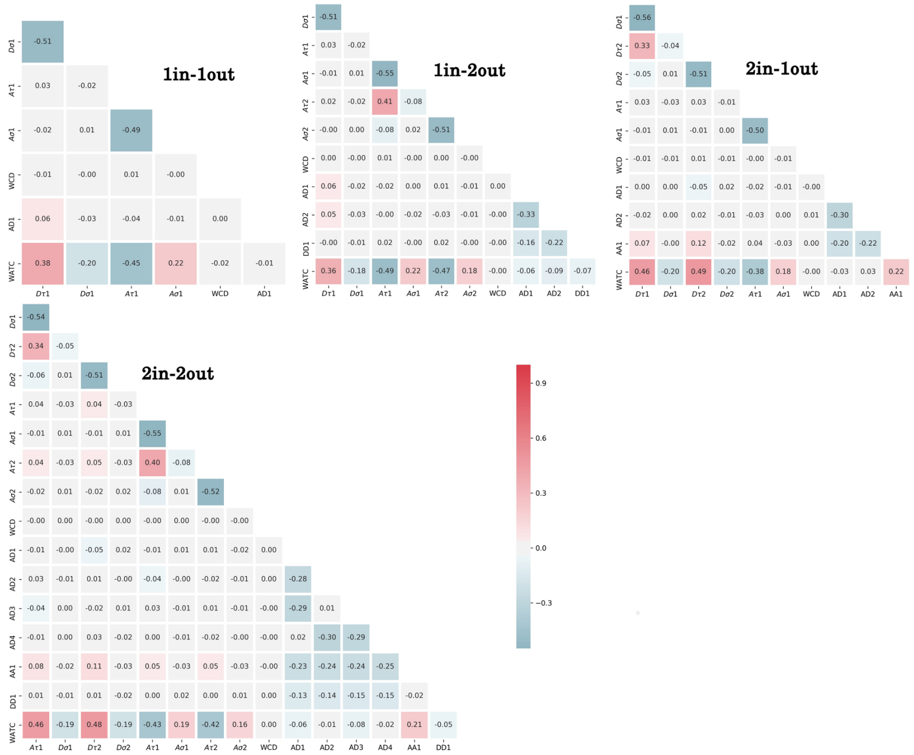

Supplementary Figure 12: Correlation matrix (using Pearson coefficients) for the second layer between the different geometrical features considered and reported in Figure 9 of the main text (Panel A and B), the distance from the Willard Chandler interface (WCD) and the water molecules total charge (WATC).

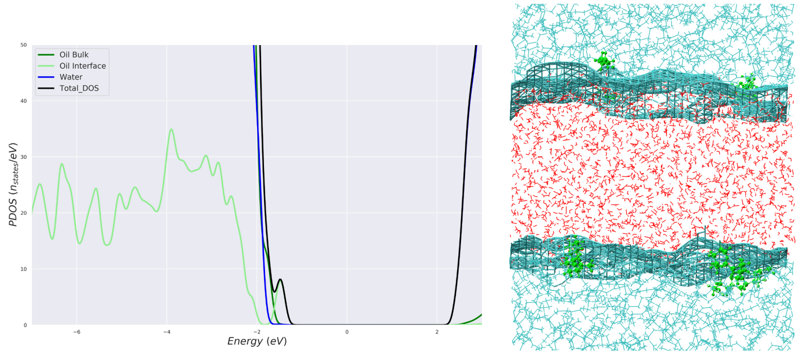

Supplementary Figure 13: Representation of the water-oil interface edge of the valence band (EVB) electronic structure. The LDOS in the left panel is obtained considering the states belonging to the oil molecules at the interface (distance from the WCI < 5.0 Å) in the bulk and to water phase. This frame is characteristic of the fluctuations where the EVB is mainly comprised by states belonging to the negatively charged oil. The right panel highlights the molecules that characterize the EVB.

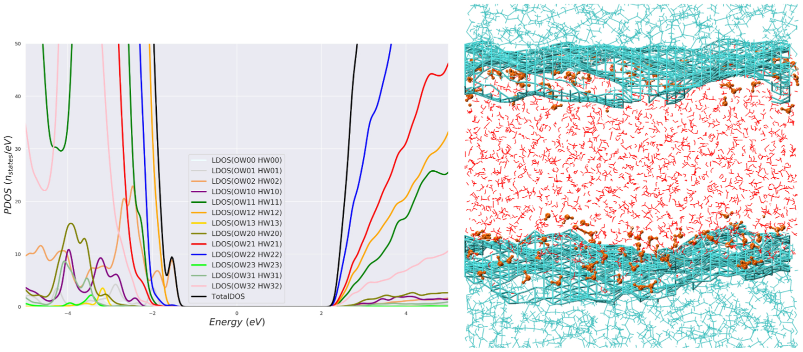

Supplementary Figure 14: Representation of the water-oil interface edge of the valence band (EVB) electronic structure. The LDOS in the left panel is obtained considering the states belonging to the different types of water coordination species. This frame is characteristic of the fluctuations where the EVB is mainly comprised by states belonging to the water phase. The right panel highlights the molecules that characterize the EVB.

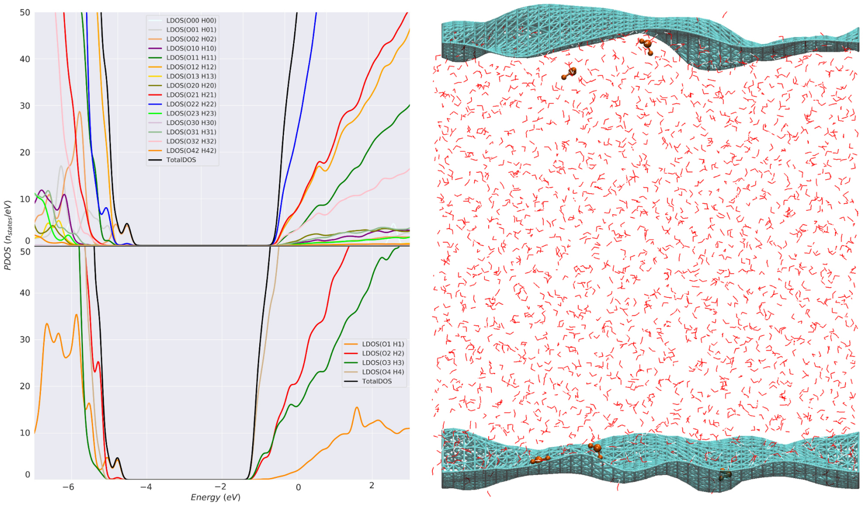

Supplementary Figure 15: Sample representation of the water-air interface edge of the valence band (EVB) electronic structure. The Local Density of States (LDOS) in the top-left panel is obtained by projecting the states for the different water coordination species. The one in the bottom-left Panel is obtained considering the projection for the water molecules belonging to the different layers defined starting from the Willard Chandler Interface (WCI) and moving towards the bulk. The water molecules belonging to the EVB in both these projections are highlighted in orange in the right panel.

Supplementary Tables

In the main text we discussed the distributions associated with the different water molecules as a function of proximity to the interface. We noted that there were subtle differences in the charge distributions such as the average charge and their variance. The tables below show the various moments of the charge distributions for both the air-water and oil-water systems for the four layers defined in the main text.

Supplementary Table 1: First moment of the layer-by-layer charge distributions for the different water molecules coordination types. The upper table values refers to Figure 4 in the main text, the lower one to Figure 6. Where values are not present it was not possible to calculate a reliable distribution due to small population.

**Water-Air**				
**2in-1out**	**2in-2out**	**1in-2out**	**1in-1out**	**1in-0ut**
0.02258	-	-0.02074	-0.00163	0.03183
0.02933	-0.00279	-0.02836	-0.00049	0.02728
0.02419	0.00270	-0.02291	-0.00020	0.02555
0.02146	0.00262	-0.02249	-0.00085	0.02024

**Water-Oil**				
**2in-1out**	**2in-2out**	**1in-2out**	**1in-1out**	**1in-0ut**
0.02522	0.00613	-0.01465	0.00144	0.03077
0.02583	0.00235	-0.02223	0.00187	0.02603
0.02320	0.00205	-0.02337	-0.00202	0.02184
0.02197	0.00240	-0.02209	-0.00235	0.02152

Supplementary Table 2: Standard deviation of the layer-by-layer charge distributions for the different water molecules coordination types. The upper table values refers to Figure 4 in the main text, the lower one to Figure 6. Where values are not present it was not possible to calculate a reliable distribution due to small population.

**Water-Air**				
**2in-1out**	**2in-2out**	**1in-2out**	**1in-1out**	**1in-0ut**
0.01456	-	0.01488	0.01773	0.01277
0.01381	0.01756	0.01367	0.01844	0.01318
0.01347	0.01919	0.01500	0.01893	0.01874
0.01564	0.02002	0.01531	0.01822	0.01435

**Water-Oil**				
**2in-1out**	**2in-2out**	**1in-2out**	**1in-1out**	**1in-0ut**
0.01275	0.01489	0.01037	0.01187	0.01526
0.01069	0.01188	0.01120	0.01125	0.01370
0.01150	0.01257	0.01108	0.01076	0.01399
0.01069	0.01151	0.01152	0.01380	0.01947

Supplementary Table 3: Third moment (skewness) of the layer-by-layer charge distributions for the different water molecules coordination types. The upper table values refers to Figure 4 in the main text, the lower one to Figure 6. Where values are not present it was not possible to calculate a reliable distribution due to small population.

**Water-Air**				
**2in-1out**	**2in-2out**	**1in-2out**	**1in-1out**	**1in-0ut**
0.25000	-	-0.09425	-0.44885	0.35494
0.58257	0.58187	-0.64743	0.68383	0.45288
-0.57637	0.73239	0.55627	0.63588	0.30624
0.60343	0.83287	-0.72084	0.65483	0.57244

**Water-Oil**				
**2in-1out**	**2in-2out**	**1in-2out**	**1in-1out**	**1in-0ut**
0.46575	0.55057	0.36443	0.45573	0.47957
0.60669	0.73138	-0.65285	0.62376	-0.39135
-0.52276	0.53996	0.54428	-0.57293	0.38563
0.68631	0.72445	-0.68475	-0.57808	0.36502

Supplementary Table 4: Fourth moment (kurtosis) of the layer-by-layer charge distributions for the different water molecules coordination types. The upper table values refers to Figure 4 in the main text, the lower one to Figure 6. Where values are not present it was not possible to calculate a reliable distribution due to small population.

**Water-Air**				
**2in-1out**	**2in-2out**	**1in-2out**	**1in-1out**	**1in-0ut**
-1.05362	-	-1.40517	-0.93984	-0.99748
-0.81209	-0.71283	-0.59665	-0.74065	-0.93704
-0.81895	-0.46109	-0.72361	-0.75306	-1.02918
-0.50922	-0.17544	-0.48052	-0.72546	-0.82289

**Water-Oil**				
**2in-1out**	**2in-2out**	**1in-2out**	**1in-1out**	**1in-0ut**
-0.81047	-0.94490	-1.09710	-0.82414	-1.01062
-0.88452	-0.72043	-0.82789	-0.86420	-0.07771
-0.73295	-0.36562	-0.70142	-0.92262	-1.98505
-0.78374	-0.73049	-0.78603	-0.91706	-1.09483

Supplementary Discussion

Water/Air and Water/Oil tests.

One of the essential factors that controls the charge transfer at the interface is the asymmetry in hydrogen bonding in terms of local coordination defects. Supplementary Figure 3 shows the distribution of total charge obtained for 1in-2out, 2in-1out and the canonical 2in-2out water molecules highlighting how the charge transfer phenomenon is reproduced when using different nuclear configurations from a more accurate potential like mb-POL.

Similar to our results for the air-water interface, the charge transfer from the water to the oil is observed also with VV10 functional, IH and NBO charge schemes (as seen in Figure 8 of the main text and Supplementary Figure 4). The scatter plots in Supplementary Figure 5 show as before, that the correlation between the DDEC charges with BLYP+D2 are in good agreement with those obtained with rVV10 as well as the other charges schemes. For the tests run on the oil-water clusters that were used for the energy decomposition analysis the top panels of Supplementary Figure 6 and 7 show the results obtained considering the charge on all atoms in the cluster comparing both BLYP/B3LYP and BLYP/MP2. These results clearly show that the charges obtained with BLYP are in good agreement with more accurate electronic structure methods. The bottom panels of Supplementary Figure 6 and 7 shows the net total charge on the decane molecule when it is negatively charged - again, we see that the predictions of BLYP are consistent with that obtained with B3LYP and MP2 indicating that the charge transfer from water to oil is a robust result.

Comparison with classical potentials.

Supplementary References 7 and 8 both use an upper and lower boundary of 0.02e (-0.02e) as a value to describe the charge transferred along a hydrogen bond. In our charges distributions we found, especially for the second layer (that gives the negative charge oscillation), larger charges than those used in this work. The left most panel of Supplementary Figure 8 shows the distribution of the charges in the second layer underlying the previous point.

In order to compare the different schemes we recomputed the charge contributions coming from individual coordination defects (middle panel of Supplementary Figure 8) and the total charge in the second layer (right most panel of Supplementary Figure 8) using the fixed upper boundary of Jungwirth et al.[7] ($\pm 0.02e$) to we obtain from our simulations. The analysis shown in Supplementary Figure 8 highlights how an increment of the average charge transfer of $\sim$50% on each H_2O molecule (and the contributions from the other species that are small but non negligible) in addition to other important factors such as the differences in the shape of the distributions of different species (Supplementary Table 3-4) and small but important differences in the average between opposite species (i.e. 1in-2out, 2in-1out) leads to a net charge in the layer (Figure 1 rightmost panel) that is much bigger with respect to the one obtained applying a averaged scheme as performed in Supplementary Ref. 7 This delicate interplay between charges and populations of difference species has to be carefully considered to get the right net charge for each layer - in our scheme, it is naturally accounted for by solving the quantum mechanical problem of the electrons. Regarding Supplementary Ref. 8 that CT-FLEX model is based to a charge transfer contribution built on the FLEX scheme[9]. In the FLEX scheme, water molecules are kept neutral and in Supplementary Ref. 8 the author adds a charge transfer contribution that is modulated via *two distance-dependent functions that smoothly go from zero to one.* This contribution is then multiplied for the ab-initio derived valued of 0.02e and redistributed on the basis of equations 6 and 7 (see Supplementaty Ref. 8) between the water molecules atoms. While this nuanced treatment of the charge transfer is more physical, the 0.02e (-0.02e) value set as maximum (minimum) parameter still does not account for the real physics of charge transfer at the interface.

Correlation between H_2O net charge and local geometry.

In order to explore if other environmental parameters are relevant in determining the charge fluctuations on the H_2O molecules, we correlated several geometrical descriptors of the environment around the water molecules, to the magnitude of the net charge observed on each

water molecule and their distance with respect from the Williard-Chandler instantaneous surface. Some of these correlation analysis are shown below for the standard 2in-2out waters and the important defects : 0in-1out, 1in-0out, 1in-1out, 1in-2out and 2out-1in (where the population is big enough to obtain a proper correlation). Our manuscript is rather long already so we will present a summarised version of the pictures/notes to follow.

Supplementary Figures 9, 10, 11, 12 show the correlation between different geometrical parameters (see Figure 9 of the main text (Panel A and B),), the total charge on each H_2O molecule (WATC) and its distance from the Willard-Chandler distance (WCD). The geometrical descriptors chosen have been previously reported in Supplementary Ref. 10 and 11. These descriptors were chosen since tetrahedrality[12,13] and Local Structure Index[14] struggled to capture the geometry of the interfacial water HB network. In fact due to the strained configurations present at the water/air boundary the significance hold by these measurements become unclear. The proton transfer coordinate (PTC) in elliptical coordinates (τ) shows a very strong negative correlation with respect to the central H_2O WATC for those water molecules accepting an HB from it (i.e. $A_\tau 1$, $A_\tau 2$). A strong positive correlation is instead observed for waters molecules donating an HB to the central H_2O (i.e. $D_\tau 1$, $D_\tau 2$). These results shows that the more the proton is shared (equivalent to smaller τ) between the molecules accepting an HB and the central H_2O the higher is the charge transferred to it making WATC more negative (this behaviour confirms the same trend observed in bulk water as showed in Supplementary Ref. 11). In the case of $D_\tau 1$ and $D_\tau 2$ the same trend stands however the charge is transferred from the central water to the other H_2O molecules making it more positive hence we observe a positive correlation.

Passing to the angular descriptors we observe the positive correlation between WATC and the angle defined by the central water molecule, $A_\tau 1$ and $A_\tau 2$. Conversely, negative correlation is observed for the angle between the central H_2O, $D_\tau 1$, $D_\tau 2$. These relations seems to suggest that the bigger is the angle formed by the central water molecule and the H_2Os accepting an HB from it the more positive is WATC. This effect could be ascribed to a

more effective alignment of the bond dipoles that results in a bigger charge transfer from the central H_2O to its coordinated counterparts. Similar reasoning but with the opposite sign can be applied for the molecules donating an HB to the central H_2O. A trend regarding the other angular parameters is generally difficult to find.

At last we consider the correlation of WCD with WATC and the geometric descriptors. Supplementary Figure 9, 10, 11 and 12 imply that it is not possible to clearly draw a direct correlation between WATC and WCD for each water molecule. It is also difficult to individuate trends between WCD and the geometrical factors considered. Nonetheless we notice that Supplementary Figures 9 and 10 show for the H_2O molecules in the first two layers a weak positive correlation of WCD with respect to the different geometrical descriptor. This relation could hint at the role of the surface vicinity in determining the strained configurations that lead to increased charge transfers at the interface.

Electronic characterization of the water-air, oil-water interfaces.

As introduced in the main text we examined the projected density of states (PDOS) of water (and oil) molecules residing in the negatively charged layer at the surface of water and found that they contribute substantially to the valence band. Supplementary Figure 13 and 14 show the contribution of both negatively charged water and oil molecules to the edge of the valence band. Supplementary Figure 15 present instead the case for the water/air interface, here the defects at the surface are about 0.5 eV higher in energy than the mostly tetrahedral ones in the bulk. As previously hinted this is indicates an uncanny similarity between the anionic defect, the hydroxide ion, and defects in neutral water.

Supplementary References

(1) Reed, A. E.; Curtiss, L. A.; Weinhold F. Intermolecular interactions from a natural bond orbital, donor-acceptor viewpoint. *Chem. Rev.* **1988**, *88*, 899-926.

(2) Vydrova, O. A and Van Voorhis, T. Nonlocal van der Waals density functional: The simpler the better. *J. Chem. Phys.* **2010**, *133* 244103

(3) Goedecker, S., Teter, M., and Hutter, J. Separable dual-space Gaussian pseudopotentials. *Phys. Rev. B* **1996**, *54*, 1703–1710.

(4) VandeVondele, J. and Hutter, J. Gaussian basis sets for accurate calculations on molecular systems in gas and condensed phases. J. Chem. Phys. **2007**, *127* 114105.

(5) Guidon, M.; Hutter, J. and VandeVondele, J. Auxiliary density matrix methods for Hartree–Fock exchange calculations. *JCTC* **2010** *6*, 2348–2364.

(6) Del Ben, M.; Hutter, J.; VandeVondele, J. Electron correlation in the condensed phase from a resolution of the identity approach based on the gaussian and plane waves scheme. *JCTC.* **2013**, *9*, 2654-2671

(7) R. Vácha, O. Marsalek, A. P. Willard, D. Bonthuis, R. Netz, and P. Jungwirth. Charge transfer between water molecules as the possible origin of the observed charging at the surface of pure water. *J Phys. Chem. Lett.*, **2012**, *3*, 107-111.

(8)Ł. J. Alexis, and R. W. Steven. The effects of charge transfer on the properties of liquid water journal. *J. Chem. Phys.*. **2011**, *134*, 184507. (9) C. D. Wick. Hydronium Behavior at the Air–Water Interface with a Polarizable Multistate Empirical Valence Bond Model. *J. Phys. Chem. C*, **2012**, *116*, 4026-4038

(10) S.Shin and A.P. Willard. Three-Body Hydrogen Bond Defects Contribute Significantly to the Dielectric Properties of the Liquid Water–Vapor Interface. *J. Phys Chem. Lett.*, **2018**, *9*, 1649-1654.

(11) C. Schran, O. Marsalek and T. E. Markland. Unravelling the influence of quantum proton delocalization on electronic charge transfer through the hydrogen bond. *Chem. Phys. Lett.*, **2017**, *678*, 289-295.

(12) P.L. Chau and A. J. Hardwick. A new order parameter for tetrahedral configurations. *Mol. Phys.*, **1998**, *93*, 511-518.

(13) J. Errington, and P. G. Debenedetti. Relationship between structural order and the anomalies of liquid water. *Nature*, **2001**, *409*, 318-321.

(14) B. Santra, R. A. DiStasio Jr,. F. Martelli, and R. Car. *Mol. Phys.*, **2015**, *113*, 2829-2841.
